# 1523. Impact of Antiretroviral Therapy (ART) on All-Cause Mortality in HIV from 1999 to 2020 in the United States

**DOI:** 10.1093/ofid/ofad500.1358

**Published:** 2023-11-27

**Authors:** Monique A Prince, Min-Choon Tan, Min-Xuan Tan, E’ebony Prince, Rick Nicholas, Hamid Shaaban, Jihad Slim

**Affiliations:** Saint Michael's Medical Center, Newark, New Jersey; Saint Michael's Medical Center, Newark, New Jersey; Monash University, Melbourne, Victoria, Australia; St. George's University, True Blue, Saint George, Grenada; University of Technology, 237 Old Hope Road, Kingston, Jamaica; St. Michael Medical Center, Newark, New Jersey; Saint Michael’s Medical Center, Newark, NJ, USA, Newark, New Jersey

## Abstract

**Background:**

Over the last two decades, Antiretroviral Therapy (ART) has evolved dramatically in the United States (U.S.) from multi-tablet regimens (MTR) to single tablet regimens (STR) and from Non-Nucleoside Reverse Transcriptase Inhibitor (NNRTI) and Protease Inhibitor (PI) based regimens to integrase inhibitor (INI) based ART. This study sought to determine the trends in all-cause mortality over time as ART trends changed.

**Methods:**

We queried the Centers for Disease Control and Prevention's Wide-Ranging Online Data for Epidemiologic Research (CDC WONDER) database and performed serial cross-sectional analyses of national death certificate data for all-cause mortality with comorbid HIV from 1999 to 2020. HIV diseases (ICD-10 B20-B24, O98.7, R75) were listed as the contributing cause of death. We calculated age-adjusted mortality rates (AAMR) per 1,000,000 individuals. Additional analyses were performed to categorize the underlying causes of death by organ systems. The study period was further stratified into three groups when specific drug regimens were more prevalent: 1999-2007 (MTR), 2008-2014 (STR) and 2015-2020 (INI).

**Results:**

In the 22-year study period, 251,759 all-cause mortalities with comorbid HIV in the United States were identified. The most common cause of death was infectious diseases (84.0%, N=211,438), followed by neoplasms (5.3%, N=13,264) and diseases of the cardiovascular system (3.9%, N=9,797). The AAMR in HIV was also highest in infectious diseases at 3.12 per 1,000,000 individuals, followed by neoplasms (0.18 per 1,000,000 individuals), diseases of cardiovascular system (0.12 per 1,000,000 individuals), diseases of respiratory system (0.05 per 1,000,000 individuals) and diseases of gastrointestinal system (0.04 per 1,000,000 individuals). Further analysis of infectious related causes showed significantly decreased mortality trends from 134,726 (MTR) to 69,450 (STR) and 47,583 (INI).

**Figure 1:**
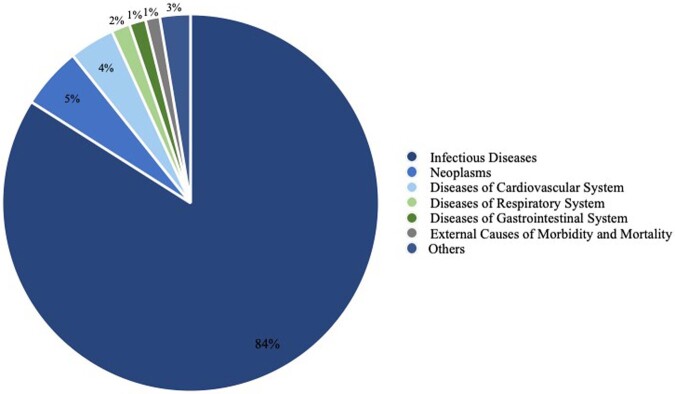
Demonstrates all-cause mortalities with comorbid HIV in the United States from 1999-2020

**Conclusion:**

More than 80% of deaths in HIV occurred because of infectious disease over the past two decades. A decreasing trend was also seen in infectious related mortality in three different time periods. This reflects the improved tolerability and efficacy of ART over time.

**Disclosures:**

**Jihad Slim, MD, FACP**, ViiV Healthcare: Advisor/Consultant|ViiV Healthcare: Grant/Research Support

